# C-756-05. Patterns of Pediatric Burn Resuscitation Protocols Across ABA-Verified U.S. Burn Centers: A Cross-Sectional Review

**DOI:** 10.1093/jbcr/irag033.049

**Published:** 2026-04-06

**Authors:** Megan E Daniels, Lud H Eyasu, Kealani Unkel, Jatin Vemuri, Rendell Bernabe, Prabhu Senthil-Kumar, Michael J Feldman

**Affiliations:** Evans-Haynes Burn Center at the VCU Medical Center/Virginia Commonwealth University, Richmond, VA; Evans-Haynes Burn Center at the VCU Medical Center/Virginia Commonwealth University, Richmond, VA; Evans-Haynes Burn Center at the VCU Medical Center/Virginia Commonwealth University, Richmond, VA; Prisma Health/University of South Carolina School of Medicine, Richmond, VA; Prisma Health/University of South Carolina School of Medicine, Richmond, VA; Evans-Haynes Burn Center at the VCU Medical Center/Virginia Commonwealth University, Richmond, VA; Virginia Commonwealth University, Richmond, VA

## Abstract

**Introduction:**

Resuscitation of pediatric burn patients presents unique challenges due to age-dependent physiology, a higher surface area-to-mass ratio, and distinct fluid requirements. Although the American Burn Association (ABA) verification process aims to ensure consistent standards of burn care, pediatric resuscitation practices remain variably defined. This study aimed to quantify protocol differences among ABA-verified centers to delineate areas of consensus and heterogeneity.

**Methods:**

We conducted a cross-sectional review of ABA-verified burn centers providing pediatric care. Protocols were obtained through direct communication with institutional leadership. The extracted data includes the formulas used for initial and maintenance fluid calculations, the choice of resuscitative fluids, and the metrics applied to guide fluid adjustments. Information on adjunctive therapies and the use of decision-support platforms was also collected.

**Results:**

Protocols were obtained from 67.4% (29 of 43) of ABA-verified burn centers providing pediatric care. Lactated Ringer’s (LR) was the initial fluid in all protocols. The initial fluid rate followed the pediatric ABA Consensus Formula (3 mL/kg/hr) in 62.1% of centers, while 17.2% used the Parkland formula (4 mL/kg/hr), and 20.7% employed other approaches. For maintenance fluids, LR with dextrose was used in 59.3%, LR without dextrose in 22.2%, 5% Dextrose with 0.45% Sodium Chloride in 14.8%, and 5% Dextrose with 0.9% Sodium Chloride in 3.7% of centers. The “4-2-1” rule guided maintenance fluid rates in 78.3% of centers, while others used varied strategies. Urine output (UOP) was the primary metric for titration in all protocols, with targets of 1.0 mL/kg/hr in 22.2%, 1.0–1.5 mL/kg/hr in 7.4%, 1.0–2.0 mL/kg/hr in 7.4%, and 30–50 cc/hr in 7.4% of centers. Additionally, 40.7% of centers stratified UOP targets by weight, and 3.7% by age. Albumin and fresh frozen plasma (FFP) were included in 62.1% and 24.1% of protocols, respectively. Decision-support tools were rarely used, appearing in only 10.3% of centers.

**Conclusions:**

Considerable variability exists in pediatric burn resuscitation protocols across centers, particularly in formula selection, choice of resuscitative fluids, and the use of colloid or fresh frozen plasma (FFP). Although urine output monitoring is universally employed, differences in threshold targets and adjunctive strategies highlight the lack of uniformity. These findings underscore the need for consensus-building and the development of pediatric-specific guidelines to promote standardization and optimize outcomes.

**Applicability of Research to Practice:**

This review characterizes national practice patterns in pediatric burn resuscitation and identifies areas of convergence and divergence. These findings may inform the development of standardized, evidence-based approaches tailored to the pediatric population.

**Funding for the Study:**

N/A.

